# Effectiveness of a school-based physical activity intervention on overweight and obesity among children and adolescents in Pakistan

**DOI:** 10.1371/journal.pone.0317534

**Published:** 2025-02-24

**Authors:** Moazzam Tanveer, Ejaz Asghar, Georgian Badicu, Alexios Batrakoulis, Umar Tanveer, Serge Brand, Matheus Santos de Sousa Fernandes, Luca Paolo Ardigò

**Affiliations:** 1 School of Physical Education and Sport Training, Shanghai University of Sport, Shanghai, China; 2 Department of Physical Education and Sport Sciences, Health Services Academy, Islamabad, Pakistan; 3 Department of Physical Education and Special Motricity, Transilvania University of Brasov, Brasov, Romania; 4 Department of Physical Education and Sport Science, University of Thessaly, Trikala, Greece; 5 Department of Mass Communication, University of Lahore, Lahore, Pakistan; 6 Center for Affective, Sleep and Stress Disorders, Psychiatric Clinics of the University of Basel, Basel, Switzerland; 7 Division of Sport Science and Psychosocial Health, Department of Sport, Exercise and Health, Faculty of Medicine, University of Basel, Basel, Switzerland; 8 Sleep Disorders Research Center, Kermanshah University of Medical Sciences (KUMS), Kermanshah, Iran; 9 Center for Disaster Psychiatry and Disaster Psychology, Psychiatric Clinics of the University of Basel, Basel, Switzerland; 10 Keizo Asami Institute, Federal University of Pernambuco, Recife, Pernambuco, Brazil; 11 Department of Teacher Education, NLA University College, Oslo, Norway; Universiti Malaya, MALAYSIA

## Abstract

**Background:**

Childhood obesity poses a significant public health challenge, yet effective school-based physical activity (PA) interventions remain scarce, especially in Pakistan. There is a lack of data assessing the impact of such interventions on obesity and related health outcomes in Pakistani school children.

**Methods:**

This study aimed to design and implement a school-based intervention targeting multiple levels of the socio-ecological model to increase physical activity and reduce the prevalence of overweight and obesity in Pakistani youth. Conducted from October 2022 to January 2023 in Lahore, Pakistan, the 12-week, non-randomized controlled trial involved 1,200 students from eight schools, with four schools (n = 570) in the intervention group and four (n = 630) in the control group. Primary outcomes included changes in body mass index (BMI), waist circumference, and the prevalence of overweight/obesity, measured through anthropometric assessments. Secondary outcomes involved alterations in moderate-to-vigorous physical activity duration. Descriptive statistics, Chi-square tests, general linear mixed models, and repeated measures ANOVA were used for analysis.

**Results:**

The intervention showed significant improvements across various socio-ecological levels. Intrapersonal factors saw a reduction in fast food consumption from 11.9% to 7.9% (F_(1,1198)_ = 90.39, *p* < 0.001; η² = 0.074) and an increase in physical activity frequency from 11.9% to 39.6% (F_(1,1198)_ = 465.25, *p* < 0.001; η² = 0.028). Screen time decreased from 27.0% to 7.4% (F_(1,1198)_ = 219.83, *p* = 0.015; η² = 0.15), and normal sleep duration increased from 44.6% to 71.8% (F(_1,1198)_ = 242.73, *p* < 0.001; η² = 0.16). At the interpersonal level, parental involvement in encouraging sports and providing financial support for sports activities significantly increased. School-level factors also showed positive changes, including improved sports facilities and equipment access. Community-level factors revealed increased opportunities for physical activity and a more supportive community environment. The intervention group’s BMI change (−0.06 ± 0.07 kg·m²) significantly differed from the control group’s (0.19 ± 0.09 kg·m²).

**Conclusions:**

This study demonstrates the effectiveness of a multi-level intervention in boosting physical activity and addressing obesity among Pakistani school-aged children, supporting the implementation of similar school-based interventions.

## Introduction

Physical inactivity and obesity among children and adolescents have emerged as significant public health concerns globally [1], as they have adverse effects on children’s and adolescents’ health, well-being and overall development [1,2]. Regular physical activity improves physical health and mental well-being [3,4]. Indeed, engaging in physical activity has been shown to offer numerous health benefits [5], including reducing the risk factors for chronic diseases, combating obesity and helping school-aged children maintain a healthy weight [6,7]. Previous studies have indicated an inverse relationship between physical activity participation and obesity [8], as well as the risk of metabolic and cardiovascular diseases among school-aged children [9–12]. However, the levels of physical activity among children and adolescents are alarmingly low: approximately 81% of adolescents worldwide reported being physically inactive in 2016 [13]. To explain such low physical activity rates, various factors such as sedentary behaviour, lack of access to recreational facilities, urbanisation, technological advancements, cultural norms, socioeconomic status and environmental factors have been identified [14,15]. For the present study, we focused on the social-ecological model.

The social-ecological model [9,16] provides a framework for understanding the critical factors influencing physical activity participation. Recognising the urgent need to address the obesity epidemic, particularly in affluent urban populations of developing countries like Pakistan, WHO Sustainable Development Goals 2030 [3]. The social-ecological model was implemented to improve physical activity levels by addressing intrapersonal factors (e.g., motivation, knowledge), interpersonal factors (e.g., social support, peer influence), organisational factors (e.g., workplace wellness programs, school physical education curriculum), community factors (e.g., access to recreational facilities, walkable neighbourhoods) and policy-level factors (e.g., zoning laws, public health initiatives [9,12,17]). Interventions based on the social-ecological model have the potential to enhance physical activity levels [9] significantly. Intrapersonal-level characteristics, such as attitude, self-efficacy and motivation, have been identified as factors potentially impacting physical activity participation [12]. Positive attitudes have been associated with increased daily steps and engagement in recreational sports [9,16,18]. Recent studies have highlighted the favourable relationship between students’ attitude and leisure-time physical activity [2,10,19]. Motivation to engage in physical activity is considered a conceptually and practically crucial component [10–12]. Creating environments that promote and support physical activity is vital in encouraging school-aged children and adolescents to be more active [12,20]. Physical education has long been linked to increased physical activity and lower body mass index among children and adolescents [12,21]. Therefore, there is a need for intervention studies that address these factors comprehensively. Interpersonal factors, including nonverbal interactions, have also been found to influence physical activity behaviour. Parental, peer and teacher support have strong correlations with physical activity promotion [16,20]. Parents can play a crucial role in supporting their children’s physical activity by encouraging their participation, monitoring their sporting endeavours, exposing them to various activities, engaging in physical activity with them and teaching them how to engage in sports or be physically active [21–23]. Researchers must understand the underlying assumptions of the ecological model to develop studies and interventions that effectively capture the pathways and interactions among the various factors contributing to overweight and obesity in school-aged children and adolescents [16,24].

Taking into account the identified research gaps, including the absence of comprehensive school-based physical activity intervention programs in Pakistan and the limited attention given to children and adolescents in this context, this study addresses the urgent need for effective strategies to combat overweight and obesity [10]. Previous interventions in Pakistan have primarily focused on psychological and extracurricular physical activities in schools, lacking the multifaceted approach required to address this growing public health concern. Therefore, this study aims to evaluate the effectiveness of a school-based physical activity intervention that incorporates multiple components, including structured physical education, promotion of healthy lifestyles, increased physical activity sessions, creation of a supportive environment, and active parental involvement [9,25]. The primary research question is whether such an intervention significantly reduces the prevalence of overweight and obesity among children and adolescents in Pakistan. Secondary questions examine how the intervention impacts physical activity levels and sedentary behavior, whether gender-based differences exist in its effectiveness, the associations between changes in physical activity levels and BMI, and the role of environmental and contextual factors such as school infrastructure and family support. It is hypothesized that the intervention will lead to a significant reduction in overweight and obesity prevalence, increase moderate-to-vigorous physical activity, and improve BMI and waist circumference, with better outcomes observed in schools with enhanced infrastructure and higher parental involvement [25]. This comprehensive framework seeks to provide evidence-based insights into designing and implementing effective interventions for improving the health of Pakistani youth.

The study addresses gaps in research by implementing a school-based multilevel intervention in Pakistan to combat low physical activity, high body mass index (BMI), and overweight/obesity in children and adolescents. Guided by the social-ecological model, the intervention targets various factors, including intrapersonal (e.g., individual behaviors and attitudes towards physical activity), interpersonal (e.g., peer and family support), organizational (e.g., school policies promoting physical activity and healthy eating), and community levels (e.g., access to safe recreational spaces). This holistic approach aims to promote physical activity and improve health by addressing knowledge, attitudes, social support, school policies, facility access, and community support systems. Through this strategy, the study seeks to provide insights into effective interventions for combating childhood obesity in Pakistan and contribute to evidence-based strategies for promoting healthy lifestyles among school-aged children and adolescents.

## Materials and methods

### Study design

**Fig 1:**
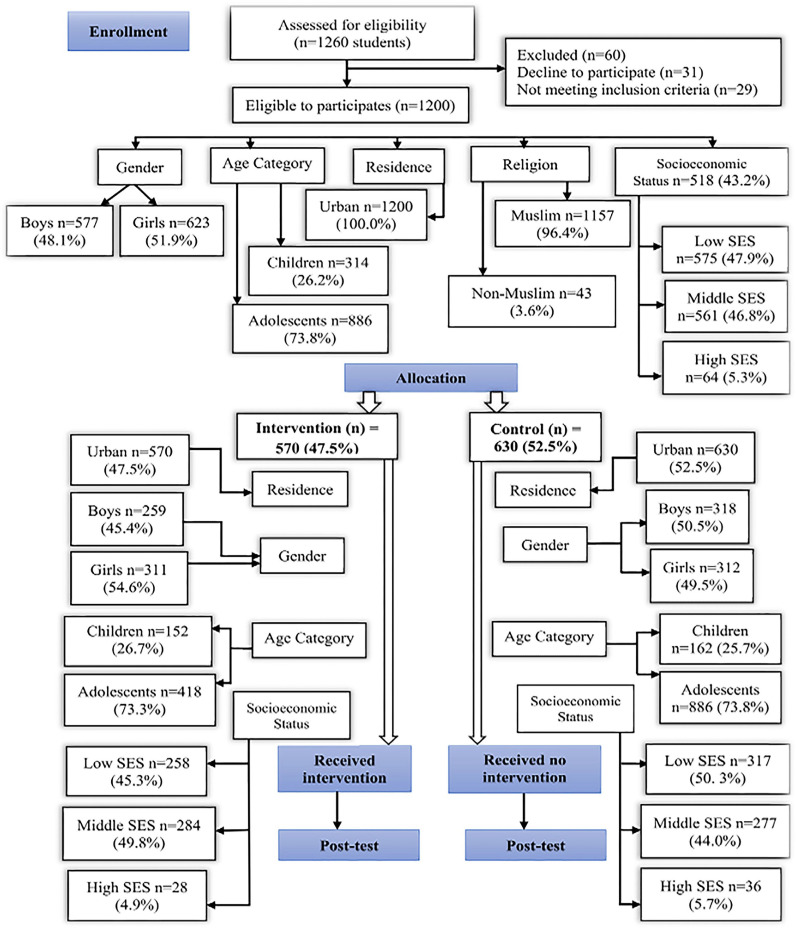
Study design.

The study employed a non-randomized controlled trial with cluster sampling to evaluate the impact of a 12-week school-based physical activity intervention on physical activity levels and overweight/obesity in children and adolescents. Conducted from October 2022 to January 2023 in eight public schools in Lahore, Pakistan, four schools were assigned to the intervention group and four to the control group. Fig 1 presents the study design and procedures, including school allocation, participant recruitment, pre-test and post-test data collection, and the intervention timeline, with the post-test conducted 12 weeks after baseline. This visual aid offers a clear summary of the study’s design and the sequence of activities.

### Setting and participants

This study targeted school-aged children and adolescents in Pakistan, aged 9 to 17, a critical developmental period. Eight schools were selected from Lahore, the capital of Punjab, which is known for its ethnic diversity and urban significance. The selection process involved cluster sampling, where schools were grouped into control and intervention categories. The Punjab School Education Department (https://sis.punjab.gov.pk/) provided a comprehensive list of schools across the region, which facilitated the random selection of schools. To ensure a diverse and representative sample, the schools were matched on key characteristics, including sex (boys and girls), age categories (9–11 years and 12–17 years), religion (Muslim and Non-Muslim), residence (urban), and socioeconomic status (low, middle, and high SES). This matching process helped to balance potential confounders and strengthen the study’s methodology by ensuring that any observed effects could be more confidently attributed to the intervention rather than underlying demographic differences. By distributing the schools equitably across the intervention and control groups, the study aimed to mitigate biases and provide more reliable results regarding the effectiveness of the school-based physical activity intervention. The study’s findings offer potential insights for analogous urban areas confronting similar health issues, enhancing the comprehension and efficacy of school-based interventions tailored to specific contexts. Enrolment took place during May 2022.

All participants or their parents or guardians (in case of participants under the age of 16) were informed about the study and provided written consent. The study was approved by the Institutional Ethics Committee the School of Physical Education and Sport Training of Shanghai University of Sport (Ethics Audit Section 2022 No. (388) X 02/01/2022). Approval to conduct the study was also obtained from the Punjab School Education Department (Approval Code: PU180220-633357) and the principals and teachers of the participating schools.

The study utilized pre- and post-test surveys conducted before and after a 12-week intervention in October 2022 and January 2023, respectively. It targeted children (ages 9 to 11) and adolescents (ages 12 to 17) from various educational levels in Pakistan. Eight schools were selected, evenly divided between boys’ and girls’ schools, representing primary, middle, secondary, and higher secondary levels. Students in grades 1 to 3 and those with physical disabilities were excluded. Initially, 1,260 students were selected, with 60 (4.76%) withdrawals, resulting in a final sample of 1,200 students (95.25%). Of these, 570 (259 boys, 311 girls) were in the intervention group, and 630 (423 boys, 207 girls) were in the control group.

### Overweight and obesity

The students’ overweight and obesity status was determined using BMI, calculated by adjusting their weight and height measurements to the nearest 0.1 kg and 0.5 cm, respectively [2,10,19]. Trained rescue professionals collected accurate anthropometric height and weight measurements in the classroom. In assessing overweight and obesity status, the study utilised the World Health Organization (WHO) child growth reference from 2007 [6,10,20]. Overweight was defined as a BMI greater than one standard deviation (SD) above the reference mean. In contrast, obesity was defined as a BMI greater than two SD above the reference mean. These criteria align with international standards and allow meaningful comparisons across populations. To ensure the appropriateness of the reference values, the growth reference tables covered the age range from 5 to 19 years. This enabled the assessment of overweight and obesity in the selected school grade cohorts, which included primary, middle, secondary and higher secondary schools [6,9,10]. The measurements were taken with students wearing appropriate clothing and assuming standing. The measurement of waist circumference followed standard protocols and guidelines [26]. This additional measure allowed for a comprehensive body composition assessment and provided valuable information related to central obesity [16].

### Instruments for the study

The study gathered data using self-reported questionnaires administered to students, with the design based on methodologies from previous international studies [2,6–10,12,16–20,26–33]. Initially available in English, the second official language of Pakistan, the questionnaires were also read aloud in Urdu, the local language, to ensure inclusivity for participants from lower grades. This approach facilitated comprehension and maximized participation, regardless of language proficiency or grade level. A serial number system, based on students’ IDs, was used for administering the questionnaires. The same ID was assigned to students in both the pre-test and post-test, enabling intrapersonal tracking and comparison of responses over time. This method allowed for the collection of comprehensive data on obesity and related health outcomes, offering valuable insights into the intervention’s effectiveness.

#### Social-ecological factors.

In this study, social-ecological factors were treated as independent variables, with data collected through a 7-day recall method from school-aged children, adolescents, their parents, and schools at two distinct time points: pre-intervention (October 2022) and post-intervention (January 2023). Although the specific questions asked during data collection are not provided, they were designed to capture information relevant to the study’s objectives. The consistent administration of these questions at both time points allowed for the assessment of changes or trends in intrapersonal factors over the intervention period, thus enabling an evaluation of its effectiveness. The 7-day recall method was chosen to capture recent behaviors and habits while minimizing recall biases associated with longer time frames.

***Intrapersonal level:*** Demographics. The questionnaire used to gather demographic information was designed based on the protocols established in previous studies [9–12,16,20]. It included relevant questions to capture demographic characteristics such as age, gender, ethnicity and other pertinent information. The questionnaire asked about a person’s sex (boy or girl), age in years (9 to 17 years), age category (child 9–11 years or adolescent 12–17 years), religion (Muslim or non-Muslim), place of residence was all from urban areas, school-type public schools, education level (primary 4–5 grads, middle 6–8 grads, secondary 9–10 grads or higher secondary 11–12 grads) and family income (in Pakistani currency [Rupee], monthly: 1 ÷ 90000 low income, 90000 to 200000 middle income, > 200000 high income [2,9,10,19,27,32]). The questionnaire’s test-retest reliability was assessed and it showed a Cronbach’s alpha of 0.96, indicating good reliability. These demographic factors were selected based on a similar survey conducted by Mushtaq et al. in Pakistan in 2011 [6,10,20]. The most recent estimates from this region suggest a Cronbach’s alpha for test-retest reliability of 0.78 [10,19,20,27].

Fast-food. Data on fast-food consumption were collected from school-aged children and adolescents using 7-day recall surveys. The questions were consistently framed, such as: “During the past 7 days, on how many days did you eat food from a fast-food restaurant, such as McDonald’s, KFC, Pizza Hut, Subway, AFC, or Rahat?” The reliability coefficient for the fast-food consumption questions was 0.81, indicating good internal consistency. Response options included “less than once,” “once or twice,” and “three or more times a week” [19,20,32].

Physical activity. The questions were consistently based on examples from previous studies [9–12,27], such as: (1) “How many days did you engage in physical activity for at least 60 minutes each day over the past seven days?” The reliability coefficient for this question was 0.84. Response categories included 0 = no days, 1 = one day, 2 = two days, 3 = three days, 5 = five days, 6 = six days, and 7 = seven days. These were grouped into categories: “less than twice or twice,” “twice or four times per week,” and “more than four times per week,” with a reliability coefficient of 0.82 [10,27]. This scale showed acceptable reliability with a Cronbach’s α of 0.89. (2) “During the past week, how many days did you walk or ride a bike to school?” The reliability coefficient was 0.77 [10,25]. (3) “How many days per week did you attend physical education (PE) class during the current academic year?” The reliability coefficient for this question was 0.86 [10,25]. The combined physical activity behavior was calculated by summing the scores from these three items, with higher scores indicating better physical activity levels [9,12]. The questions used were adapted from the 2016 Pakistan Global School-based Student Health Survey (GSHS) Questionnaire [32] and the study by Mushtaq et al. [10]. To assess whether participants met the moderate-to-vigorous physical activity (MVPA) requirement, we followed the guidelines from the WHO, as well as Canadian and Chinese 24-Hour Movement Guidelines, which recommend a minimum of 60 minutes of MVPA daily [27,34].

Sedentary behavior. The following questions were consistently used to assess screen time behavior: (1) “On school days and weekends, on average, how long did you watch TV over the previous week?” with a reliability coefficient of 0.72 [10,27]. (2) “How many hours per day did you spend using a cell phone, tablet, or other mobile electronic devices on school days and weekends?” with a reliability coefficient of 0.79 [10]. (3) “During the previous week, on school days and weekends, how many hours per day did you spend sitting in front of computers, laptops, and other devices for tasks?” with a reliability coefficient of 0.83 [10]. (4) “How much homework do you have each day after school and on weekends?” with reliability coefficients of 0.78 and 0.73 [10,19]. The scale for assessing sedentary behavior demonstrated acceptable reliability (Cronbach’s α = 0.81), and the combined score was calculated by summing responses to the four questions. A lower score indicated better screen time behavior. Categories for screen time were defined as (a) less than one hour, (b) one or more hours, and (c) three or more hours per day. The assessment was aligned with the WHO, Canadian, and Chinese 24-Hour Movement Guidelines, which recommend limiting screen time to two hours or less per day [2,10,27,34].

Sleeping behaviour. The following questions were consistently used in the survey: (1) “From Monday through Friday, what hour do you usually go to sleep?” with a reliability coefficient of 0.83 [27]; (2) “From Monday to Friday of the previous week, what time did you wake up?” with a reliability coefficient of 0.73 [27]; (3) “What hour do you often go to bed on Saturday and Sunday nights?” with a reliability coefficient of 0.74 [28]; and (4) “On weekends, particularly Saturday and Sunday, what time do you get up in the morning?” with a reliability coefficient of 0.83 [28]. Participants reported their typical sleep patterns on weekdays and weekends. Sleep duration was estimated by subtracting the “go to bed” time from the “get up” time, with the reliability coefficient for this estimation also being 0.83 [28]. The combined sleep behavior score was calculated by summing the scores from the four survey items. Sleep duration was categorized based on the 24-Hour Movement Guidelines from the WHO, CDC, Canada, and China for children aged 6–12, as follows: (1) < 9 hours (“short” sleepers), (2) 9–12 hours (“regular” sleepers), and (3) > 12 hours (“long” sleepers). For adolescents aged 13–18, the categories were: (1) < 8 hours (“short” sleepers), (2) 8–10 hours (“regular” sleepers), and (3) > 10 hours (“long” sleepers). The average sleep duration was calculated as [(weekday sleep duration × 5) + (weekend sleep duration × 2)]/ 7 [28]. The sleep-related questions were adapted from the 24-Hour Movement Guidelines and previous studies, including Chen et al. [27], Stefan et al. [28], and the 2016 Pakistan Global School-based Student Health Survey (GSHS) [32].

***Interpersonal level:*** Data were collected from parents at two time points: pre-test (October 2022) and post-test (January 2023). Parents were asked the following questions: (1) “Do you encourage your children’s participation in sports?” (reliability coefficient 0.81) [25,30]. (2) “Do you and your family participate in sporting activities for fun (e.g., swimming, cycling)?” (reliability coefficient 0.82) [25,30]. (3) “Would you offer financial assistance for sports-related activities (e.g., paid training, equipment)?” (reliability coefficient 0.77) [25]. The overall Cronbach’s alpha for these items was 0.96 in our study, indicating high internal consistency compared to 0.82 in previous studies [30]. Parents responded with “1” for “Yes” and “2” for “No,” reflecting their support for their children’s participation and family engagement in physical activities.

***School level factors:*** Data were collected from students at two time-points: pre-test before the 12-week intervention in October 2022 and post-test in January 2023. Students responded to questions regarding school-level factors, with answer options of “Yes” (1) and “No” (2). These questions assessed aspects of the school environment related to physical activity and sports participation. The questions included: (1) “Does the school provide students with access to sports facilities?” (reliability coefficient = 0.87) [25], focusing on playground satisfaction, availability of physical education teachers, and the effective use of funds for sports facilities. (2) “Does the condition of the school’s sports equipment meet the requirements for daily PE?” (reliability coefficient = 0.74) [25,31], evaluating the suitability of sports equipment for daily PE. (3) “Is the school sports venue open to students free of charge on weekends?” These items’ internal consistency, measured by Cronbach’s alpha, was 0.91 [31].

***Community level:*** Parents’ perceptions were assessed at two time points: pre-test before the 12-week intervention in October 2022 and post-test after the intervention in January 2023. The questionnaire focused on community factors related to the neighborhood’s safety for children and their access to physical activity options. Parents responded with “Yes” (1) or “No” (2). The first question, “What choices for physical activity are in your immediate surroundings?” (reliability coefficient = 0.73) [25,33], aimed to assess the availability and variety of nearby physical activity opportunities. The second question, “How about a life study of students supported by the community?” (reliability coefficient = 0.73) [33], sought to gauge parents’ perceptions of community support for promoting a healthy lifestyle among students, including initiatives that encourage physical activity.

#### Intervention study.

A 12-week multicomponent physical activity intervention was conducted among Pakistani students to address overweight and obesity. The intervention group participated in enhanced physical education classes, tailored extracurricular activities for overweight/obese students, encouragement of physical activity at home, and health education lectures for both students and parents. Principles such as Supportive, Active, Autonomy, Fair, and Enjoyable were incorporated to ensure effectiveness. These efforts aimed to promote active lifestyles, healthy habits, and a supportive school and home environment, ultimately improving the students’ health outcomes.

***Physical activity components:*** The physical activity component of the intervention program was designed according to the American College of Sports Medicine guidelines, which recommend a minimum of 60 minutes of moderate-to-vigorous physical activity (MVPA) daily for children and adolescents. The program included three main elements: enhancing physical education classes, extracurricular activities for overweight/obese students, and promoting family physical activity with parental involvement [9,12,16]. Schools were expected to improve the content, intensity, and duration of physical education to meet the recommended activity levels. Primary school students (Grades 4 and 5) primarily engaged in stretching, sprinting, endurance running (e.g., 50 m shuttle run), rope jumping, and light throwing. Secondary and higher secondary students participated in endurance running (e.g., 1000 m for boys, 800 m for girls), cycling, kabaddi, long jumping, and basketball [12,16]. The intensity and duration of MVPA were evaluated based on students’ fitness levels, categorized as “good,” “medium,” or “poor.”

Physical education instructors monitored students’ engagement, coordination, and self-perceived exhaustion to assess class intensity. To maintain quality, PE experts supervised and guided instructors, ensuring that intensity levels were met. For overweight and obese students, PE teachers organized extracurricular sessions, including aerobics, walking, jogging, cycling, and body movement exercises targeting the trunk, hips, and legs. Although participation was not mandatory, these students were encouraged to engage in MVPA at least three days a week, aiming for 30 minutes daily. Intensity levels were monitored, and active participation was encouraged [9,12,16].

Additionally, students were assigned home workouts, including exercises like jogging or rope jumping for primary students. They maintained exercise diaries to track intensity and duration. Those who met the requirements received praise and recognition for their efforts [16].

***Health education lectures:*** The intervention program included comprehensive health education lectures aimed at educating students on obesity prevention and healthy lifestyle choices. Topics covered included the causes and effects of childhood obesity, the role of BMI in screening for overweight/obesity, healthy eating, physical activity promotion, and the importance of adequate sleep. These lectures provided students with practical guidance and encouraged healthier habits, such as increasing fruit and vegetable consumption, reducing sedentary time, and ensuring sufficient sleep. Educational materials and newsletters were distributed to students and parents to create a supportive environment for implementing healthy behaviors both at home and in school [16].

Health education lectures were conducted for both students and parents to emphasize the importance of physical activity, healthy eating habits, and overall lifestyle choices that contribute to maintaining a healthy body weight. These educational sessions aimed to promote a supportive home environment by involving both groups. As part of the intervention, parents received two newsletters containing information about the potential effects of excessive screen time, strategies for increasing physical activity, reducing sedentary behavior, and promoting recreation at home. The newsletters also provided tips on how to establish regulations without causing conflict and included their child’s preliminary fitness test results. The intervention encouraged parents to supervise and support their children’s healthy lifestyle choices, focusing on healthy eating (e.g., increasing fruit and vegetable consumption), physical activity (e.g., engaging in sports together), and limiting sedentary behavior (e.g., screen time of less than two hours per day). Additionally, the intervention addressed sleep recommendations (9–12 hours for children aged 6–12 years, 8–10 hours for adolescents aged 13–18 years) and the importance of avoiding unhealthy behaviors, such as alcohol consumption and tobacco use. The educational material was delivered through indirect methods, such as student diaries, and direct methods, including a WhatsApp group, to ensure effective communication and engagement with parents.

***Outcome measures:*** The study utilised various outcome measures to assess the effectiveness of the intervention. Primary outcome measures included changes in BMI, waist circumference and the prevalence of overweight/obesity, which were determined through anthropometric measurements and BMI categorisation. Secondary outcome measures encompassed alterations in the duration of MVPA, quantified through self-reported data collected by physical education teachers, as well as the number of physical education classes and extracurricular activities attended by students [16]. Additionally, student attendance records were examined to evaluate engagement in the intervention program, while self-reported degrees of exhaustion, enthusiasm and coordination provided insights into students’ subjective experiences during physical activities. These measures collectively offered a comprehensive evaluation of the intervention’s impact on students’ physical health and participation in structured physical activities within the school setting [12,16].

***Process evaluation:*** The process evaluation of the school-based interventions aimed to assess the implementation and effectiveness of the intervention components. Various process measures were implemented to gather data and provide valuable insights into the intervention’s progress. This section outlines details of the evaluation process, including data collection methods, observations, meetings, and interviews. PE teachers actively monitored PE and extracurricular activities, recording attendance, fatigue and enthusiasm. The research staff tracked health education lectures, materials distribution and parent involvement through daily updates and WhatsApp communication. Various process measures, including student attendance, leadership accreditation, teacher satisfaction and parental involvement, were employed to assess intervention progress comprehensively. School-level observations, health steering committee meetings and program evaluation visits provided further insights into intervention effectiveness. At the same time, face-to-face interviews with key stakeholders offered a deeper understanding of successful intervention components and factors influencing effectiveness [9,12,16].

#### Theoretical framework.

The intervention in this study was grounded in the socio-ecological model, acknowledging the diverse influences on individuals’ behaviours and health outcomes. It aimed to enhance physical activity levels and reduce overweight and obesity among students in intervention schools, contrasting with control schools that maintained their regular curriculum without intervention [9]. Designed as a multi-level approach, the intervention targeted students, families, teachers, school policies and the physical and social environments within schools. Practical improvements in physical education, extracurricular activities for overweight/obese students and health education for students and parents were key components. By addressing these areas, the intervention aimed to foster supportive environments for physical activity and healthy behaviours both at school and at home. Recognising the significance of various factors within children’s immediate surroundings, efforts were concentrated on modifying school and family settings where children spend substantial time. At the school level, enhancements in physical education classes and opportunities for exercise were implemented, while at the family level, the engagement of parents in promoting physical activity was emphasised. Community involvement was also sought to create a supportive environment conducive to physical activity and healthy behaviours. By integrating interventions across multiple levels and involving diverse stakeholders, the intervention aimed to establish sustainable changes for the long-term health and well-being of school-aged children and adolescents [16].

### Statistical analysis

The statistical analysis was performed using SPSS v.26 (IBM, Armonk, New York, USA), with a significance level set at *p* < 0.05. Age was calculated in days by subtracting the date of birth from the date of examination. Z-scores for BMI-for-age were calculated using the WHO’s AnthroPlus software, with overweight and obesity categorized based on the WHO child growth reference (2007). Overweight was defined as a BMI-for-age z-score greater than + 1 SD, and obesity was defined as a z-score greater than + 2 SD. Waist circumference was measured in centimeters following WHO guidelines [26]. Descriptive statistics, t-tests (for continuous normal data), and Chi-square tests (for categorical data) were used to assess group differences. The intervention’s effects on outcome measures were analyzed using general linear mixed models and repeated measures analysis of variance (ANOVA). Effect sizes were calculated using partial eta squared (η²). A cut-off value of η² = 0.01, 0.06, and 0.14 was used to interpret small, medium, and large effect sizes, respectively. These statistical methods and effect size calculations provided valuable insights into the intervention’s impact on various factors.

## Results

The baseline demographic characteristics of the participants were analyzed, revealing 1,200 students with a mean age of 13.24 years (Table 1). Of these, 570 were assigned to the intervention group (mean age = 13.05 years) and 630 to the control group (mean age = 13.42 years). The sex distribution was balanced, with 259 boys and 311 girls in the intervention group and 318 boys and 312 girls in the control group (*p* = 0.081). Age category analysis showed no significant difference between groups (*p* = 0.708). Most participants were Muslim, with no significant difference in religion (*p* = 0.895). Socioeconomic status was distributed as low SES (47.9%), middle SES (46.8%), and high SES (5.3%), with no significant difference between groups (*p* = 0.125). The baseline mean BMI was 19.58 kg·m², with no significant difference between the intervention (19.35 kg·m²) and control (19.78 kg·m²) groups (*p* = 0.109). The prevalence of overweight/obesity was 34.2%, with 31.4% in the intervention group and 36.7% in the control group (*p* = 0.147). Waist circumference did not differ significantly between the intervention (mean = 67.75 cm) and control (mean = 68.18 cm) groups (p = 0.443).

**Table 1:** Baseline demographic characteristics of participants.

Characteristics	Intervention group	Control group	Total	p-value
**Sample size, n (%)**	570 (47.5)	630 (52.5)	1,200 (100)	
**Age (year, mean±SD)**	13.05* ± 2.11*	13.42* ± 2.27*	13.24* ± 2.21*	
**Sex, n (%)**				
Boy	259 (45.4)	318 (50.5)	577 (48.1)	0.081
Girl	311 (54.6)	312 (49.5)	623 (51.9)	
**Age Category, n (%)**				
Children 9–11 years	152 (26.7)	162 (25.7)	314 (26.2)	0.708
Adolescent 12–17 years	418 (73.3)	468 (74.3)	886 (73.8)	
**Religion, n (%)**				
Muslim	550 (96.5)	607 (96.3)	1157 (96.4)	0.895
Non-Muslims	20 (3.5)	23 (3.7)	43 (3.6)	
**Residence, n (%)**				
Urban	570 (47.5)	630 (52.5)	1,200 (100)	
**Socioeconomic status (SES)**				
Low SES	258 (45.3)	317 (50.3)	575 (47.9)	0.125
Middle SES	284 (49.8)	277 (44.0)	561 (46.8)	
High SES	28 (4.9)	36 (5.7)	64 (5.3)	
**BMI (kg·m** ^**−2**^ **, mean±SD)**	19.35* ± 4.72*	19.78* ± 4.49*	19.58* ± 4.61*	0.109
**Overweight/obesity, n (%)**	179 (31.4)	231 (36.7)	410 (34.2)	0.147
**Waist circumference, (mean±SD), cm**	67.75* ± 9*.63	68.18* ± *9.69	67.98* ± *9.66	0.443

Note. BMI (body mass index).

The primary outcomes, including waist circumference, BMI, and overweight/obesity prevalence, were analyzed for both the intervention and control groups, with results summarized in Table 2. Intention-to-treat analyses were performed. Chi-square tests were used to categorize overweight/obesity, while t-tests measured waist circumference and BMI. The intervention group showed a statistically significant reduction in waist circumference (−0.8 ± 0.17 cm), whereas the control group experienced an increase (0.58 ± 0.14 cm) (p = 0.001). Similarly, the intervention group had a significant decrease in BMI (−0.06 ± 0.07 kg·m^−2^), while the control group showed an increase (0.19 ± 0.09 kg·m^−2^) (p = 0.012). Overweight/obesity prevalence decreased by 2.6% in the intervention group and increased by 1.7% in the control group, with a significant difference between groups (*p* = 0.005).

**Table 2:** Changes in waist circumference, body mass index and prevalence of overweight/obesity in intervention and control groups.

Characteristics	Intervention group (n = 570) mean±SD	Control group (n = 630) mean±SD	Difference, mean (95% CI)	p-value
**Waist circumference, (mean±SD), cm**				
Pre-test,	67.75 ± 9.63	68.18 ± 9.69	−1.52 to 0.66	0,443
Post-test,	66.94 ± 9.08	68.76 ± 10.01	−2.90 to 0.72	0.001
Mean (SE) change	−0.81 (0.17)	0.58 (0.14)		
**Body mass index, kg·m** ^**−2**^				
Pre-test,	19.35 ± 4.72	19.78 ± 4.49	−0.94 to 0.09	0,109
Post-test,	19.29 ± 4.66	19.97 ± 4.66	−1.20 to −0.14	0.012
Mean (SE) change	−0.06 (0.07)	0.19 (0.09)		
**Overweight/Obesity**				
Pre-test, %	31.4	36.7		0.147
Post-test, %	28.8	38.4		0.005
Change, %	−2.6	1.7		
Change difference, %	−4.3			

Table 3 presents the social-ecological characteristics of students in the intervention and control groups at baseline and post-test, revealing significant differences after the intervention. In the intervention group, there was a marked improvement in students’ health behaviors. For example, the percentage of students consuming fast food three or more times per week decreased from 11.9% to 7.9%, while those engaging in physical activity four or more times per week increased from 11.9% to 39.6%. Additionally, screen time of three or more hours per day dropped from 27.0% to 7.4%, and normal sleep duration improved from 44.6% to 71.8%. Family involvement also increased, with more parents encouraging physical activity and providing financial support. At the school level, access to sports facilities and equipment improved, while community-level factors showed enhanced opportunities for physical activity and support. These findings highlight the broad impact of the intervention on students’ behaviors and their environments.

**Table 3:** Comparison of multilevel social-ecological indicators between intervention groups at baseline and post-test using chi-square test.

Time	Characteristics	Intervention Group (n = 570)	Control group (n = 630)	χ^2^	p-value
**Intrapersonal factors**					
**Fast food.**					
**Pre-test**	<1 once/week	374 (65.6)	382 (60.6)	7.05	0.029
	1–2 times/week	128 (22.5)	139 (22.1)		
	≥3 times/week	68 (11.9)	109 (17.3)		
**Post-test**	<1 once/week	416 (73.0)	320 (50.8)	63.15	<0.001
	1–2 times/week	109 (19.1)	204 (32.4)		
	≥3 times/week	45 (7.9)	106 (16.8)		
**Physical Activity**^a^.					
**Pre-test**	≤2 times/week	455 (79.8)	499 (79.2)	0.37	0.828
	>2 times–4 times/week	47 (8.2)	49 (7.8)		
	≥4 times–7 times/week	68 (11.9)	82 (13.0)		
**Post-test**	≤2 times/week	90 (15.8)	459 (72.9)	400.05	<0.001
	>2 times–4 times/week	254 (44.6)	67 (10.6)		
	≥4 times–7 times/week	226 (39.6)	104 (16.5)		
**Screen Time**^b^.					
**Pre-test**	≤1 hour/day	341 (59.8)	360 (57.1)	4.26	0.119
	>1 hour–2 hours/day	75 (13.2)	110 (17.5)		
	≥3 hours	154 (27.0)	160 (25.4)		
**Post-test**	≤1 hour/day	419 (73.5)	288 (45.7)	113.41	<0.001
	>1 hour–2 hours/day	109 (19.1)	171 (27.1)		
	≥3 hours	42 (7.4)	171 (27.1)		
**Sleep duration.**					
**Pre-test**	Normal sleeper	254 (44.6)	279 (44.3)	0.42	0.807
	Short sleeper	211 (37.0)	226 (35.9)		
	Long sleeper	105 (18.4)	125 (19.8)		
**Post-test**	Normal sleeper	409 (71.8)	227 (36.0)	154.84	<0.001
	Short sleeper	114 (20.0)	261 (41.4)		
	Long sleeper	47 (8.2)	142 (22.5)		
**Family factors**					
**Encourage children to participate in sport.**					
**Pre-test**	Yes	381 (66.8)	432 (68.6)	0.41	0.522
	No	189 (33.2)	198 (31.4)		
**Post-test**	Yes	460 (80.7)	368 (58.4)	69.50	<0.001
	No	110 (19.3)	262 (41.6)		
**Take part in sports activities with children.**					
**Pre-test**	Yes	194 (34.0)	227 (36.0)	0.52	0.469
	No	376 (66.0)	403 (64.0)		
**Post-test**	Yes	416 (73.0)	212 (33.7)	185.57	<0.001
	No	154 (27.0)	418 (66.3)		
**Provide funds to children for sports.**					
**Pre-test**	Yes	317 (55.6)	327 (51.9)	1.65	0.198
	No	253 (44.4)	303 (48.1)		
**Post-test**	Yes	458 (80.4)	363 (57.6)	71.56	<0.001
	No	112 (19.6)	267 (42.4)		
**School factors**					
**The school provides students with access to sports facilities.**					
**Pre-test**	Yes	343 (60.2)	360 (57.1)	1.13	0.228
	No	227 (39.8)	270 (42.9)		
**Post-test**	Yes	458 (80.4)	491 (77.9)	1.05	0.304
	No	112 (19.6)	139 (22.1)		
**The condition of the school’s sports equipment facilities to meet the requirements of daily PE.**					
**Pre-test**	Yes	292 (51.2)	341 (54.1)	1.00	0.315
	No	278 (48.8)	289 (45.9)		
**Post-test**	Yes	419 (73.5)	354 (56.2)	39.15	<0.001
	No	151 (26.5)	276 (43.8)		
**The school sports venue opens to students free of charge on weekends.**					
**Pre-test**	Yes	84 (14.7)	77 (12.2)	1.62	0.202
	No	486 (85.3)	553 (87.8)		
**Post-test**	Yes	413 (72.5)	188 (29.8)	217.39	<0.001
	No	157 (27.5)	442 (70.2)		
**Community factors**					
**Opportunities for physical activity available in community.**					
**Pre-test**	Yes	67 (11.8)	134 (21.6)	20.56	<0.001
	No	503 (88.2)	486 (78.4)		
**Post-test**	Yes	304 (54.3)	184 (29.2)	77.08	<0.001
	No	256 (45.7)	446 (70.8)		
**Children lives in a supportive community.**					
**Pre-test**	Yes	308 (54.0)	397 (64.0)	12.29	<0.001
	No	262 (46.0)	223 (36.0)		
**Post-test**	Yes	400 (71.4)	409 (64.9)	5.76	0.016
	No	160 (28.6)	221 (35.1)		

Note.

^a^Physical activity included running or jogging, cycling, housework or yard work and sports involving physical movement (see details in methods).

^b^Sedentary lifestyle included television viewing, working on computer and playing video games.

Comparing the baseline and post-test results, significant differences were observed between students in the intervention group and those in the control group regarding their social-ecological characteristics, as shown in Table 4. The interaction of time and group for all individual-level factors, including fast-food consumption (F_(1,1198)_ = 90.39, p < 0.001; η^2^ = 0.074), physical activity (F_(1,1198)_ = 465.25, p < 0.001; η^2^ = 0.028), screen time (F_(1,1198)_ = 219.83, p = 0.015; η^2^ = 0.15) and sleep duration (F_(1,1198)_ = 242.73, p < 0.001; η^2^ = 0.16), was statistically significant. These results explained 7.4%, 28.9%, 15.5% and 16.3% of the variance in PA participation, respectively. Regarding interpersonal family-level factors, significant improvements were observed in the intervention group. The factors of parents encouraging children to participate in sports (F_(1,1198)_ = 40.58, p < 0.001; η^2^ = 0.03), parents taking part in sports activities with children (F_(1,1198)_ = 114.69, p < 0.001; η^2^ = 0.08) and parents providing funds for sports (F_(1,1198)_ = 24.66, p < 0.001; η^2^ = 0.02) were statistically significant. These results accounted for 3.3%, 8.1% and 2.7% of the variance, respectively. At the school level, significant improvements were observed in school-level factors within the intervention group. The availability of sports facilities (F_(1,1198)_ = 30.69, p < 0.001; η^2^ = 0.02), equipment facilities to meet daily physical education requirements (F_(1,1198)_ = 24.69, p < 0.001; η^2^ = 0.02) and the opening of sports venues on weekends (F_(1,1198)_ = 147.02, p < 0.001; η^2^ = 0.11) were statistically significant. These results accounted for 2.5%, 2.1% and 11.7% of the variance in school-level factors, respectively. Regarding community-level factors, improvements were observed in the intervention group. The availability of opportunities for physical activity in the community (F_(1,1198)_ = 96.36, p < 0.028; η^2^ = 0.07) and the existence of a supportive community (F_(1,1198)_ = 18.11, p < 0.001; η^2^ = 0.01) were statistically significant. These results accounted for 7.3% and 1.7% of the variance in community-level factors, respectively.

**Table 4:** Effect of intervention on multilevel social-ecological indicators.

Characteristics	Time	Intervention group (n = 570) mean±SD	Control group (n = 630) mean±SD	Treatment x interaction F	p-value	Effect size ηp^2^
**Intrapersonal factors.**						
**Fast food.**	**Pre-test**	1.46 ± 0.69	1.56 ± 0.76	90.39	<0.001	0.07
	**Post-test**	1.34 ± 0.62	1.66 ± 0.74			
**Physical activity.**	**Pre-test**	1.32 ± 0.67	1.33 ± 0.69	465.25	<0.001	0.28
	**Post-test**	2.23 ± 0.70	1.43 ± 0.75			
**Screen time.**	**Pre-test**	1.67 ± 0.87	1.68 ± 0.85	219.83	<0.001	0.15
	**Post-test**	1.33 ± 0.60	1.81 ± 0.83			
**Sleep duration.**	**Pre-test**	1.74 ± 0.75	1.76 ± 0.76	242.73	<0.001	0.16
	**Post-test**	1.36 ± 0.63	1.87 ± 0.75			
**Family factors.**						
**Encourage children to participate in sport.**						
	**Pre-test**	1.33 ± 0.47	1.31 ± 0.46	40.58	<0.001	0.03
	**Post-test**	1.19 ± 0.39	1.41 ± 0.49			
**Take part in sports activities with children.**						
	**Pre-test**	1.65 ± 0.47	1.63 ± 0.48	114.69	<0.001	0.08
	**Post-test**	1.27 ± 0.44	1.66 ± 0.47			
**Provide funds to children for sports.**						
	**Pre-test**	1.44 ± 0.49	1.48 ± 0.50	24.66	<0.001	0.02
	**Post-test**	1.19 ± 0.39	1.42 ± 0.49			
**School factors.**						
**The school provides students with access to sports facilities.**						
	**Pre-test**	1.39 ± 0.48	1.42 ± 0.49	30.69	<0.001	0.20
	**Post-test**	1.19 ± 0.39	1.22 ± 0.41			
**The condition of the school’s sports equipment facilities to meet the requirements of daily PE.**						
	**Pre-test**	1.48 ± 0.50	1.45 ± 0.49	24.69	<0.001	0.02
	**Post-test**	1.26 ± 0.44	1.43 ± 0.49			
**The school sports venue is open to students free of charge on weekends.**						
	**Pre-test**	1.85 ± 0.35	1.87 ± 0.32	147.60	<0.001	0.11
	**Post-test**	1.27 ± 0.44	1.70 ± 0.45			
**Community factors.**						
**Opportunities for physical activity available in community.**						
	**Pre-test**	1.88 ± 0.32	1.78 ± 0.41	96.36	<0.001	0.07
	**Post-test**	1.46 ± 0.49	1.71 ± 0.45			
**Children lives in a supportive community.**						
	**Pre-test**	1.46 ± 0.49	1.36 ± 0.48	18.11	<0.001	0.01
	**Post-test**	1.29 ± 0.45	1.35 ± 0.47			

All individual data are available in the supplementary file S1 File.xlsx.

## Discussion

The high prevalence of overweight and obesity among children and adolescents in Pakistan calls for effective interventions aimed at increasing physical activity and reducing BMI. School-based interventions have emerged as a promising approach to promote healthier behaviours and combat the growing obesity epidemic. This discussion will focus on the potential of school-based interventions in Pakistan to increase physical activity levels and reduce BMI among children and adolescents.

The results of this study indicate that school-based interventions can effectively enhance physical activity levels and reduce BMI among children and adolescents, consistent with findings from other countries [12,35]. These positive outcomes are particularly encouraging in Pakistan, where resources and infrastructure for promoting physical activity are limited [10,36]. A key strength of the intervention was the use of the social-cological model as a theoretical framework, which addresses multiple levels of influence on health behaviors, including individual, interpersonal, organizational, and community factors [9,16,24,33]. By targeting these levels, the interventions aimed to create a supportive environment for promoting physical activity and managing weight among young individuals.

The interventions in this study incorporated several key components to enhance physical activity and reduce BMI, overweight, and obesity [16]. Physical education (PE) was recognized as essential, providing a structured opportunity for all students to engage in regular physical activity [9]. However, the study highlighted the common issue of insufficient PE provision in Pakistani schools, emphasizing the need for improvement [12,35]. By optimizing PE time and improving its quality, the interventions aimed to increase physical activity levels [37,38]. Extracurricular physical activities were also emphasized, offering additional opportunities tailored to overweight and obese children and adolescents [16]. Professional PE teachers played a vital role in supervising these activities, ensuring proper guidance and support [39].

Furthermore, the interventions incorporated a family component, recognising the influence of familial support and involvement on children’s physical activity behaviours and weight management [37,40]. By engaging parents and promoting physical activity and healthy behaviours within the family context, the interventions aimed at creating a supportive home environment that reinforces the desired behaviours. The findings of this study also emphasise the importance of community-level factors in promoting physical activity and reducing overweight and obesity [16,37,40].

The interventions aimed at improving the supportive community environment by disseminating information and resources through parent newsletters. This community-level component aimed to create awareness, provide guidance and facilitate access to resources that promote physical activity and healthy behaviours. It is important to note that treating overweight and obesity among children and adolescents is a complex task, as it involves addressing multiple factors and requires sustained behaviour change [16,20]. School-based interventions provide a promising avenue for promoting physical activity and weight management. Still, they should be part of a broader, multifaceted approach that involves collaboration between schools, families, communities and policymakers [9,41]. Long-term sustainability, ongoing support and adequate resources are critical for the success of such interventions.

This study inferred a significant effect of intervention underpinned by individual, interpersonal and organisational correlates of PA. Our study’s findings align with the previous study [9], which reported significant improvement in enhancing PA in students after the intervention. Similarly, previous research reported that those with higher motivation levels for PA generally possess higher PA levels [12,42]. This study’s results also agree with the past intervention based on exercise motivation to increase students’ PA. The researchers confirmed the importance of exercise motivation in improving PA behaviour [43].

Both theory and research provide evidence that increased motivation leads to higher levels of physical activity (PA) [9,16]. The present study found that an intervention based on attitudes toward exercise significantly influenced healthy eating, physical activity, sedentary behavior, and sleep patterns among school-aged children and adolescents. Consistent with these findings, previous studies have reported a strong association between exercise attitudes and PA in this demographic [12,41,44]. This study contributes to the literature by emphasizing the importance of addressing individual-level factors such as motivation and attitudes in interventions promoting PA and healthier behaviors. These results highlight the need to incorporate strategies that enhance motivation and foster positive attitudes toward exercise in future programs. Based on these findings, future interventions in Pakistan should focus on techniques that boost motivation for PA and cultivate positive exercise attitudes, such as incorporating motivational strategies, creating supportive environments, and promoting the benefits of PA and healthy behaviors.

The focus of this study was to examine the impact of social support for physical activity (PA) from family, peers, and teachers on PA behavior. Consistent with prior research, the findings highlight that social support plays a pivotal role in enhancing PA among children and adolescents, both in school and at home [9,16]. Integration of PA within family, peer, and teacher contexts significantly positively influenced PA levels in school children and adolescents [36,45]. These results align with earlier intervention studies, which found that social support-based interventions effectively increased PA levels in school settings [16]. Moreover, a recent study in Pakistan demonstrated that family support significantly predicted PA behavior among students [9]. A systematic review on social-ecological factors influencing PA among adolescents similarly showed that support from peers, parents, and teachers significantly explained PA participation [46]. The present study’s findings also correspond with previous research emphasizing the importance of positive parental relationships in fostering PA engagement among youth [47]. However, the present study’s results contradict those of Kiyani et al., who found no significant association between parental and teacher support and PA among Pakistani students [25]. This discrepancy may stem from differences in study design, sample characteristics, or cultural context, highlighting the need for further investigation into these factors.

Numerous interventions have targeted healthier behaviors at home, including promoting nutritious meals, limiting screen time, encouraging physical play, and, in some cases, removing electronic devices from children’s bedrooms [22,48]. This study highlights the significant improvements in family-level factors, particularly parental involvement, in reducing overweight and obesity among children. These findings align with previous studies that have reported similar outcomes [46]. A key takeaway from this study is the crucial role of family commitment in changing behaviors and improving children’s health. The strong association between parental involvement and increased physical activity underscores the importance of parental support in promoting physical activity and reducing obesity. This research provides a foundation for future studies on the interpersonal factors influencing physical activity among school-aged children and adolescents, emphasizing the need to explore the mechanisms behind these factors and develop strategies to enhance social support and parental engagement in promoting physical activity.

Organizational factors at the school level are crucial in addressing childhood obesity and related health issues [49]. Schools offer favorable settings for health promotion and provide opportunities to implement strategies for preventing overweight and obesity [41]. This study focused on three key areas: physical activity, nutrition behavior, and physical education, which are essential for promoting healthy habits among students [50]. Some school-based interventions have integrated these elements to encourage healthy behaviors [51]. Consistent with previous studies, the findings suggest that interventions targeting physical education improvement, extracurricular activities for overweight/obese students, and health education for students and parents were effective in promoting physical activity, laying a foundation for future research [16,51]. Similar outcomes have been reported, where participation in such interventions reduced the risk of overweight and obesity among both boys and girls [16,51].

Furthermore, this study explored various organisational factors that significantly improved adolescents’ PA behavior. The provision of PA facilities, a safe PA environment, PA policies, and a supportive PA culture positively influenced adolescents’ PA, except for PA equipment. These findings are consistent with previous research conducted by other investigators [16,39]. The availability, accessibility, greening of school sports venues and promoting a sports culture atmosphere were important institutional factors in promoting PA among students [9]. These findings align with previous studies that emphasize the importance of the school environment in school-based interventions to enhance PA [12,16]. It is worth noting that a recent empirical research study and systematic review by Stylianou et al. found inconclusive associations between school PA policies and PA levels [9]. This discrepancy may be due to differences in the age of the study samples or other contextual factors. Additionally, organisational factors may influence PA behaviour indirectly through social support or personal factors, highlighting the need for further investigation in this regard.

Community-based interventions targeting overweight and obesity have employed strategies such as family workshops, after-school programs and employee health initiatives [18]. However, the success of these initiatives has been mixed and many face limitations similar to those of previous programs. Previous research by Swinburn et al. reported a 6% decrease in children’s overweight and obesity as a result of community-based interventions [37]. Some interventions have successfully reduced sedentary behaviours, such as decreased TV viewing time [22]. However, sustaining the intervention beyond the initial funding period and achieving long-term results have proven challenging for many community-based programs. Swinburn et al. identified two main difficulties in these interventions: maintaining community participation and ensuring access to necessary resources [27,37]. In line with the literature, the present study also found that community-level factors played a significant role in improving the environment for physical activity and reducing overweight and obesity among school-aged children and adolescents.

The intervention in this study aimed to enhance community-level factors through parent newsletter distribution, fostering a supportive environment for physical activity. Significant improvements were observed in reducing overweight and obesity among the target population. While community-based interventions show promise, addressing challenges identified in previous research is crucial, particularly ensuring sustained participation and access to resources for long-term success. Engaging community members and securing sustainable funding are essential for continued effectiveness. This study highlights the impact of school-based interventions in increasing physical activity and reducing BMI, overweight, and obesity among Pakistani children and adolescents. By incorporating a socio-ecological model, the intervention targeted individual, interpersonal, organizational, community, and policy factors, creating a supportive environment for healthier behaviors. Future research should refine these interventions to effectively address the growing overweight and obesity issue in low-income countries like Pakistan.

### Strengths

The strengths of this study are crucial to its relevance in childhood obesity and physical activity interventions. Grounded in a social-ecological model, it considers the multifaceted influences on children and adolescents, ensuring a comprehensive understanding of the issue. Conducting the intervention in Pakistan adds diversity to intervention settings, enhancing the applicability of findings to similar contexts. The study evaluates strategies to increase physical activity and reduce BMI, overweight, and obesity, addressing the growing childhood obesity problem in Pakistan. Based on previous research and recommendations, the intervention methods have a higher potential for effectiveness. Engaging parents and promoting physical activity at home further supports in-school interventions, boosting the likelihood of success. Lastly, the study fills a significant research gap in Pakistan, contributing valuable insights to the existing literature on childhood obesity in this context. These strengths underscore the study’s importance and provide a foundation for future research and interventions.

### Limitations

This study has several strengths, but it is essential to acknowledge limitations that may affect the interpretation and generalizability of the findings. These limitations include small sample sizes, reliance on a single informant group from urban schools, the use of BMI alone for measurement, the non-equivalent control group design, the short intervention duration, and the need for further exploration of the intervention’s long-term effectiveness. Future research could address these limitations by conducting regional or provincial-level studies, implementing long-term interventions with follow-up assessments, including a broader age range of students, and expanding sample sizes with representative sampling. Additionally, triangulating data sources, assessing various obesity-related measures, and evaluating the durability and stability of interventions over time would enhance the knowledge base. By considering these research perspectives, future studies can further explore effective interventions for addressing childhood obesity and promoting healthier lifestyles among children and adolescents.

## Conclusions

In conclusion, this study’s multi-component physical activity intervention has demonstrated significant improvements in BMI levels, physical activity duration and various health behaviours among children and adolescents. The intervention effectively promoted physical activity engagement both within the school environment and at home, leading to positive changes in individual, interpersonal, school and community factors. Specifically, the intervention resulted in decreased fast-food consumption, increased physical activity duration, reduced screen time and improved sleep patterns at the individual level. At the interpersonal level, parental engagement in sports activities increased, highlighting the importance of parental support in promoting physical health. At the school level, enhancements in facilities and resources for physical activity and improvements in physical education classes were observed, emphasising the role of supportive school environments. At the community level, the intervention leveraged community resources and support to promote physical activity among participants. These findings underscore the effectiveness of school-based interventions in addressing childhood obesity and promoting a physically active lifestyle. They have implications for public health policies and interventions aimed at combating childhood obesity and fostering healthy behaviours among children and adolescents.

Future research should focus on evaluating the long-term sustainability of school-based physical activity interventions, including follow-up assessments to determine whether improvements in BMI, physical activity levels, and health behaviors are maintained over time. Additionally, exploring the scalability and adaptability of these interventions in different regions and cultural contexts within Pakistan, as well as assessing their cost-effectiveness, would provide valuable insights for broader implementation. Investigating the impact of integrating technology, such as mobile apps or online platforms, to enhance engagement and support sustained behavior change could further strengthen interventions. Furthermore, understanding how cultural and societal factors, particularly related to gender and physical activity norms, influence intervention effectiveness, using frameworks like Self-Determination Theory (SDT), could offer key insights. Future studies should also explore the role of teacher and parental behaviors in supporting psychological needs during interventions, assessing whether SDT-informed strategies lead to greater adherence and improved outcomes.

## Supporting information

S1 File Pre- and post-data file.
(XLSX)

S1 Appendix A. The Intervention Components and Contents. (PDF)

S1 Questionnaire Student, parental, and school survey questionnaires. (DOCX)
