# Supplementary material for: Effectiveness of a school-based physical activity intervention on overweight and obesity among children and adolescents in Pakistan
Source: PLoS One. 2025 Feb 24;20(2):e0317534. doi: 10.1371/journal.pone.0317534 (PMC11849862; doi:10.1371/journal.pone.0317534)
Supplement: S1 Appendix — (PDF) [file pone.0317534.s002.pdf]

Appendix A (Supportive, Active, Autonomous, Fair and Enjoyable) teaching principles.

Principles	Strategies
Supportive – Sessions conducted in a supportive environment	1. Publicly recognize all students' effort, learning, achievements, and improvement.<br>2. Provide feedback on student effort, process and progress (not results).<br>3. Identify and manage inappropriate student behavior (e.g., teasing, over-competitiveness).<br>4. Promote positive social interactions between students.
Active – Sessions involve a high level of active time	1. Use small-side games, circuits and tabloids to maximize participation.<br>2. Ensure school sports equipment is plentiful and developmentally appropriate.<br>3. Monitor in-class physical education.<br>4. Use student leaders to set-up games and activities.
Autonomous – Sessions involve elements of choice and opportunities for graded tasks	1. Ensure that tasks incorporate multiple challenge levels, and give students the freedom to select level of difficulty.<br>2. Provide students with opportunities to create and modify rules and activities.<br>3. Provide students with opportunities for leadership roles.<br>4. Encourage students to assess their own skill performances (e.g., detect and correct their own errors).
Fair – Sessions provide all students with an opportunity to experience success	1. Ensure tasks are not dominated by the most competent students.<br>2. Modify the tasks to increase the opportunity for success (i.e., make the goals bigger, reduce the number of defensive players, alter the equipment used, revise the task rules).<br>3. Ensure students are evenly matched in competitive activities.<br>4. Acknowledge and reward participation and good sportsmanship.
Enjoyable – Sessions are designed to be enjoyable and engaging for all students.	1. Include a wide variety of games and activities.<br>2. Provide engaging and age-appropriate tasks.<br>3. Avoid boring and repetitive activity.<br>4. Don't use exercise or activity as punishment.

Appendix B Components and content of multilevel health interventions.

Intervention component	Dose	Aims	Activity	Form of Intervention	Material provided	Mode of delivery
**Teachers**						
**Teacher professional**	2 × 4-hour workshops	Provide instruction.<br>General encouragement.<br>Plan social support or social change.<br>Provide information about behavior health link.	Throughout the research period, teachers attended two professional development workshops (pre- and mid-program). The workshops were given a rationale for the program, a summary of the intervention techniques (such as program elements and behavioral messages), and an explanation of the intervention's conceptual framework.	Motivation in school sport.	PPT, health newsletter.<br>Colored leaflets	Face to face.<br>WhatsApp group.
**Parents**						
**Parental education/ awareness**	2 × newsletters	Provide feedback on performance.<br>Plan social support or social change.<br>Provide information about behavior health link.<br>Behavior contract.	Newsletters were sent to the parents of study participants with information on the potential effects of youth screen use, techniques for increasing physical activity and decreasing physical inactivity, recreation in the home, and advice on how to apply regulations without causing conflict. Also, they received their child's preliminary fitness test results.	To encourage and supervise their children to have a healthy lifestyle. Healthy eating (increasing consumption of vegetables and fruits), physical activity (sports activities together). Sedentary lifestyle (Screen time must be less than 2 hours per day), Sleeping (children 6-12 age 9–12 h, Adolescents 13-18 age 8–10 h), and Unhealthy behavior (“never” drink alcohol and never use any tobacco).	Health newsletters	Indirect method (student diary)
**School children**						
**Morning Exercise**	6 x 15 min sessions.	Enhanced school sport sessions.<br>Motivation in Morning Exercise.	Before entering the classroom each morning, schoolchildren and adolescents participated in physical activities including aerobics.	6 x 15 min sessions.<br>Students was participated in six Morning physical activity mentoring sessions.	Model or demonstrate	Face to face.<br>Physical education teacher
**Strengthen the concept of physical activity and Exercise attitude**	3 x 20 min	Providing introduction to PA and its benefits.<br>changing attitude towards doing PA by	The researcher used colored postcards or booklets to prepare the class for participation in activities as he presented on the benefits of PA for health and wellbeing during a group session in PE theory class. Putting A into Practice in PE Practical Class and During Lunch.	Informational approach<br>Behavior change-based approach.	Colored leaflets on PA health benefits PPT A group rules sheet<br>Consequences of doing PA Diary sheets for home assignment.	Face to face in class<br>Home assignments for doing activity<br><br>Group activities in the ground
**Exercise motivation Exercise self-efficacy**	6 x 15 min sessions.	Motivating them to do more and more PA enhancing their ability to do PA.	The explanation on developing self-efficacy and motivation for PA was given by encouraging students to participate in PA and improving their capacity to do PA.	Information based approach<br>Behavior change-based approach.	PPT, Colored leaflets<br>Positive climate for increasing PA<br>Assignment sheet	Face to face in class<br>Home assignments for doing activity

			The students engaged in PA throughout breaks and during the PE practical instruction. Students were encouraged to become more active and to prevent remaining still and doing nothing.			Practicing PA in class and playgrounds
**Teachers and peers support**	6 x 15 min sessions.	Encouraging students to participate in PA and reducing inactivity by making students play with their peers in pairs and in groups supervise and guide their activities.	The teacher-led activities in this session were carried out in the classrooms and on the playground. Instructors encouraged their students to stand up more often and practice PA. Appreciate the students' active participation. They emphasized the benefits of physical activity for health. Instructors guided students in reaching their physical activity goals by demonstrating how to participate in productive physical activity. Students were taught to cooperate when doing PA, respect other children's PA, and share their experiences with one another.	Information based approach<br>Behavior change-based approach<br>Discussion<br>Explanation<br>Sharing PA experience<br>Cooperation in doing PA	PPT, Colored leaflets<br>Instructions on how to perform PA<br>Positive group climate for doing PA	Face to face in class<br>Physical education teacher-led group activities in PE practical class and playground<br>Making students pairs and groups for peer activities
**Family support**	3 x 15 min sessions.	Giving awareness to family to encourage their child for doing PA, and to play with them in their free time	Asked students, "Do parents encourage students to do physical activities in their free time at school"? or the siblings play with them?<br>Posters were present with family doing PA with their children. Students was asked to do PA with their parents and siblings in their free time<br>Students were given home assignments on their diaries to instruct their siblings and parents to play with them and do PA with them.<br>The home assignments also included instructions on encouraging children to participate in PA, and provide them support for PA verbally and physically by playing with them.	Behavior change-based approach<br>Encouraging PA<br>Cooperating in doing PA	Colored leaflets<br><br>Assignment Health newsletters	Face to face in class<br>Home assignments for doing activity with their family (parents and siblings)
**School sports equipment and facilities.**	3 x 20 min sessions.	Providing sports equipment to students for practicing PA	The use of the sporting equipment was explained to the students. Introduce the school's sporting facilities Remember the specific area from images of the facilities' sporting goods. Students had access to these opportunities during PE practical class. They were given instructions on how to use them, and there were activities in the classroom and on the playgrounds. Questions and responses about facilities, equipment, and physical education.	Information based approach<br>Behavior change-based approach.	Provision of sports equipment such as rope, sport equipment<br>School facilities such as provision of playground. stadium open on Saturdays and Sundays in school.<br>Colored prints or leaflets on different PA activities with the provided material	Face to face in class<br><br>PET, Activities in class and in the playgrounds.

					Demonstration of PA and sports behavior.	
**Lunch-time physical activity mentoring sessions**	6 x 30 min sessions.	Motivation in school sport.	Six lunchtime mentoring sessions for physical activity involved students. These independent sessions entailed recruiting and training volunteers in resistance exercise with elastic tubing.	6 x 30 min sessions.<br>Students was participated in six Morning physical activity mentoring sessions.	Model or demonstrate	Face to face.<br>Physical education teacher
**Physical activity policy and culture**	3 x 20 min sessions.	To teach school PA policy to students, and explain if there was PA culture in the school.	The regulations of the PA policy and how the school is applying them were posted in the classrooms. Ten minutes, "Exercise for an hour every day," and "Promoting physical activity, 2027 Plan" are introduced.	Information based-approach<br>Behavior change-based approach	Power-point presentation<br>Colored leaflets<br>Banners	Face to face in the class<br>Perform PA according to PA policy in the school
**Call on students to participate in PA and School sports.**	6 x 10 min sessions.	To revise all activities held in previous sessions	All of the sessions were briefly reviewed during this session's conclusion, and sports-related activities were carried out both inside and outside of classrooms during breaks. Following that, students engaged in activities and exchanged and shared their experiences. Ask students about their experiences as they discuss about them.	Behavior change-based approach	Colored prints<br>Banners, Discussion	Face to face in class<br>In practical class and in the grounds
